# Novel thin-GaN LED structure adopted micro abraded surface to compare with conventional vertical LEDs in ultraviolet light

**DOI:** 10.1186/s11671-015-0885-4

**Published:** 2015-04-15

**Authors:** Yen Chih Chiang, Chien Chung Lin, Hao Chung Kuo

**Affiliations:** Institute of Lighting and Energy Photonics, National Chiao Tung University, No.301, Gaofa 3rd Rd., Guiren Dist., Tainan City, 71150 Taiwan; Institute of Photonic System, National Chiao Tung University, No.301, Gaofa 3rd Rd., Guiren Dist., Tainan City, 71150 Taiwan; Department of Photonics and Institute of Electro-Optical Engineering, National Chiao Tung University, 1001 University Road, Hsinchu, 300 Taiwan

**Keywords:** Gallium nitride, Light-emitting diode, Vertical injection, Ultraviolet, Textured surface

## Abstract

In this study, novel thin-GaN-based ultraviolet light-emitting diodes (NTG-LEDs) were fabricated using wafer bonding, laser lift-off, dry etching, textured surface, and interconnection techniques. Placing PN electrodes on the same side minimized the absorption caused by electrodes in conventional vertical injection light-emitting diodes (V-LEDs) and the current spreading was improved. The light output power (700 mA) of the NTG-LEDs was enhanced by 18.3% compared with that of the V-LEDs, and the external quantum efficiency (EQE) of the NTG-LEDs was also relatively enhanced by 20.0% compared with that of a reference device. When the current operations were 1,500 mA, the enhancements of the light output power and EQE were 27.4% and 27.2%, respectively. Additionally, the efficiency droop was improved by more than 15% at the same current level.

## Background

A wide range of applications use ultraviolet (UV) lamps as a light source. These applications, such as chemical ink curing, disease or virus inspection, and air/water purification, traditionally adapt mercury-based lamps that are not environmentally friendly. To replace these mercury-based units, nitride-based UV light-emitting diodes (UV-LEDs) have recently received considerable attention because of their light-weight, high-efficiency, and eco-friendly features [1-4]. However, currently, the traditional nitride-based UV-LEDs cannot attain extremely high efficiency. Several improvements, such as AlInGaN barriers, high-temperature grown AlN buffers, pattern sapphire substrates, and current blocking layers, involving wafer epitaxy and layer designs have been proposed [5-10]. Apart from wafer design and quality problems, the sapphire-based UV-LEDs are influenced by the poor thermal dissipation of substrates and low light extraction efficiency [11-13]. Vertical injection LEDs (V-LEDs) have been recently demonstrated as one of the most promising technologies for achieving superior brightness operation because of their excellent thermal dissipation [14-20]. In addition to thermal problems, to strengthen the light extraction efficiency, the surfaces of the V-LEDs are abraded to enable extra scattering capability [21-26]. Furthermore, the current V-LED metal-contact design affords extra absorption because of layouts and materials. In this study, a novel layout design combined with innovative fabrication processes facilitated placing p and n contact metals on the same side of the main LED lighting area; this effectively eliminated the aforementioned electrode absorption loss and maintained the advantages of vertical bonding architecture. In the following sections, novel thin-GaN-based ultraviolet light-emitting diodes (NTG-LEDs) that include such designs and were created through advanced processing techniques (such as wafer bonding, laser lift-off (LLO), dry etching, textured surface, and interconnection processes) are described.

## Methods

In this study, LED wafers were produced by depositing low-pressure metal-organic chemical vapor (LP-MOCVD) onto c-face (0001) 2-in.-diameter sapphire substrates. The LED structure comprised a 20-nm-thick GaN nucleation layer, 0.5-μm-thick undoped GaN layer, 2.0-μm-thick Si-doped n-type AlGaN cladding layer, unintentionally doped active region of 365-nm emitting wavelengths with six periods of InGaN-AlGaN multiple quantum wells (MQWs), 0.2-μm-thick Mg-doped p-type GaN cladding layer, and Si-doped n-InGaN-GaN short period superlattice structure.

**Figure 1:**
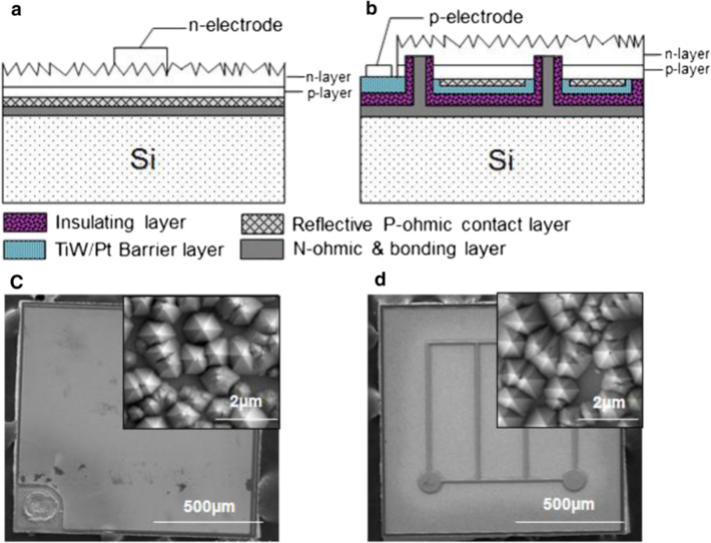
Schematic diagrams and scanning electron microscope images of devices. **(a, b)** Both configurations and **(c, d)** SEM observations include appearances and micro abraded surface morphologies (insets).

Figure 1 shows a schematic diagram of conventional V-LEDs and NTG-LEDs. The fabrication processes of the NTG-LEDs were began to define the several n-contact vias by using an inductively couple plasma (ICP) to etch through the MQW to the n-GaN by an ICP etcher. After removing the photo-resist, a highly reflective ohmic contact layer of Ni (3 Å)/Ag (2,000 Å)/Ti (300 Å)/Pt (800 Å) was deposited on the blank wafer with n-contact vias and treated by 30-min thermal annealing at 430°C. In our design, the transparent conductive layer (TCL) which usually consists of indium tin oxide (ITO) is eliminated due to the light extraction concerns and flip-chip package layout. The TCL is favorable in conventional lateral blue LED fabrication, and it provides a highly transparent and conductive window for the p-side contact. However, in our devices, the less conductivity of the p-side contact was flip-chip bonded on a metal layer; thus, the current spreading was not an issue for the p-side conductivity. Furthermore, the emission wavelength was demonstrated in the UV region, where the TCL can absorb as high as 40% of the incident photons. Based on these two assessments, the TCL configuration was not included in our design. Subsequently, a metallic barrier layer of TiW (1,200 Å)/Pt (500 Å) was deposited and covered on the previous metal layer to prevent the subsequent silver from migrating to the PN junction, leading to the short circuiting of the device. An insulating layer of Si_3_N_4_ (5,000 Å) was deposited on the entire surface through lithography and buffer oxide etchant (BOE) wet etching processes to expose the n-contact layer. Next, a Cr (300 Å)/Pt (500 Å)/Au (15,000 Å) bonding layer was evaporated to cover the gap and the entire area. The sample was then bonded onto a Cr (300 Å)/Pt (500 Å)/Au (15,000 Å)-coated n-type conducting Si substrate at 350°C for 1 h to form the flip-chip configuration illustrated in Figure 1b. The bonded samples were subsequently subjected to the LLO process to remove the sapphire substrate. A KrF excimer laser at a wavelength of 248 nm and with a pulse width of 25 ns was used to withdraw the sapphire substrate. The laser with a beam size of 0.3 mm × 0.3 mm was incident from the polished backside of the sapphire substrate onto the sapphire-GaN interface to decompose GaN into Ga and N. After removing the sapphire substrate, the samples were dipped into a HCl solution to eliminate the residual Ga from the u-GaN. Details of the LLO process are described in [17].

**Figure 2:**
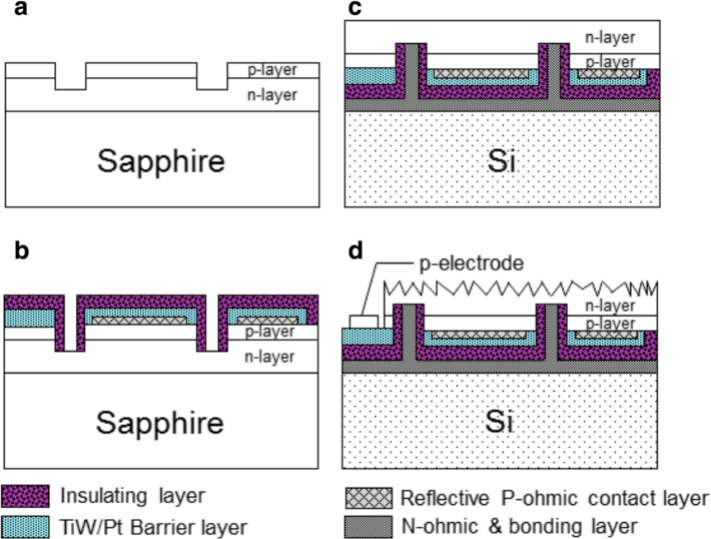
Process flows of NTG-LEDs. **(a)** Defined the n-contact area, **(b)** n-area exposed from insulating layer **(c)** transferred the structure to silicon **(d)** abraded n-surface and deposited the p-electrode.

**Figure 3:**
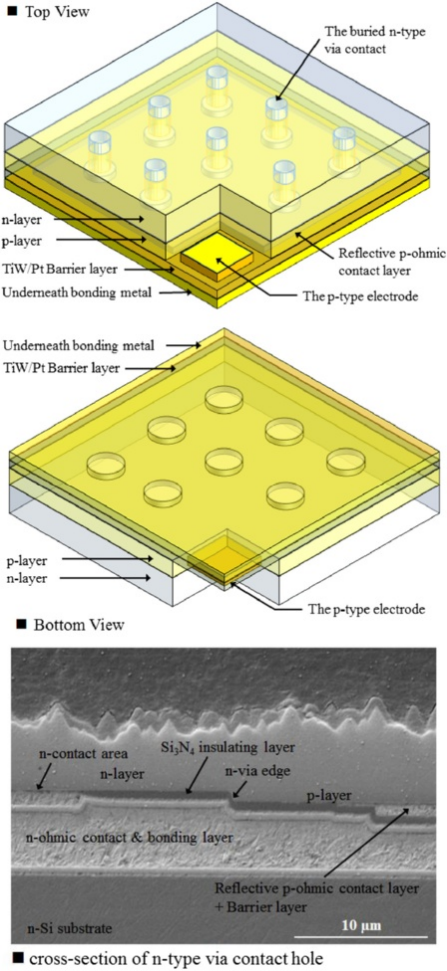
The 3-D schematic diagrams and cross-sectional SEM image of detailed n-type via contact hole.

The u-GaN was then etched away to reveal the n-AlGaN layer by using an ICP dry etcher; a square mesa (1,150 μm × 1,150 μm) was created using ICP for current isolation purposes. To improve light output power and further enhance the light extraction efficiency of the NTG-LEDs, a micrometer-range random abraded surface was formed on the n-AlGaN by using a KOH solution at 80°C for 120 s. Finally, the p-electrode contact area was created through ICP etching, and the process was completed by depositing a Ti (300 Å)/Al (1,500 Å)/Ni (1,000 Å)/Au (10,000 Å) metal electrode. Figure 1c,d illustrates the scanning electron microscope images of the V-LEDs and NTG-LEDs. The insets of Figure 1c,d illustrate that a micrometer abraded surface is created on both samples’ emission area. Figure 2 shows a brief summary of the process flow. The three-dimensional schematic structure of NTG-LED with both views (top and bottom) and cross-sectional SEM image of detailed n-type via contact hole are illustrated in Figure 3.

**Figure 4:**
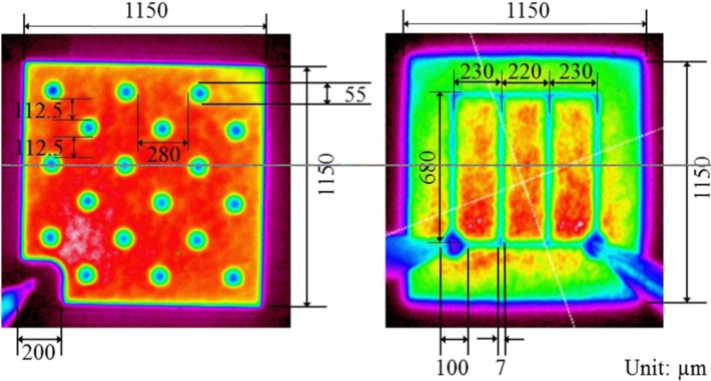
The detailed specification of the metal pattern in both samples.

Because of the considerable changes in the contact layout design, the light-emitting areas of the V-LED and NTG-LED must be compared. The metal grid width for n-electrode of the V-LED is 7 μm, and the diameter of via for n-type electrode of the NTG-LED is 55 μm. The detailed specification of the metal pattern is shown in Figure 4. According to these specifications, the percentage of shadowing effect (*R*_shadow_) is defined, and it can be expressed as follows:

1 $$ {R}_{\mathrm{shadow}}=\frac{A_{\mathrm{non}\hbox{-} \mathrm{emission}}}{A_{\mathrm{emission}}}\times 100\% $$

where *R*_shadow_ is the ratio of the shadowing effect, *A*_non-emission_ is the dark area due to the contact metal, and *A*_emission_ is the area of illumination.

The *R*_shadow_ of both devices were 3.3% (V-LEDs) and 6.3% (NTG-LEDs). The conventional V-LEDs exhibited substantial n-type metal on the top surface, whereas the n-contact layer of the NTG-LEDs was buried beneath the active region. Therefore, no obvious electrode was observed in the NTG-LEDs. As compared to V-LEDs, NTG-LEDs only sacrificed a small corner of emission area to create the p-type contact area. This feature can reduced the packaging cost from requiring two gold wires to one gold wire and lower the absorption from fewer bonding pads and gold wires. In addition, the test results indicated that the output power of the NTG samples substantially improved. Although the NTG samples had a higher shadowing factor than did the traditional V-LED, the distributed n-contact considerably facilitated the injection uniformity; thus, at the same pumping current, the overall output power of the NTG device was substantially stronger than that of the traditional V-LED. The optical and electrical characteristics were measured at room temperature by using a manual probing system featuring an integrating sphere detector and Keithley 2600 (Keithley Instruments Inc., Cleveland, OH, USA). To prevent the thermal effect during continuous DC current from causing a decrease in light-output power, *L*-*I*-*V* characteristics were measured under the pulse mode with a 2.5% duty cycle. The light intensity distribution and cross-sectional intensity profile were measured by Unice’s LED beam profile detective system (Unice E-O Services Inc., Chungli, Taoyuan, Taiwan). Firstly, both samples were operated at 1,500 mA to fix the dark level/gain/threshold settings and then gradually reduce the current to measure the field distribution at the lower level of injection.

## Results and discussion

**Figure 5:**
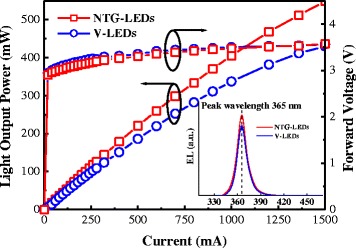
Forward voltage and light-output power as a function of current for both LEDs under pulse current injection. The inset shows the EL spectrum of the two devices.

Figure 5 (inset) illustrates the electroluminescence (EL) spectra of both devices, indicating that the peak wavelength of both devices was at 365 nm. The EL intensity of the NTG-LEDs was higher than that of the reference device. According to the *L*-*I*-*V* characteristics shown in Figure 5, the *I*-*V* curves for both devices were almost identical (approximately 3.25 V at 350 mA); this similarity indicates that the fabrication processes did not degrade the electrical properties of the devices. In addition, the *L*-*I* characteristics indicate that the NTG-LEDs demonstrated more favorable linear characteristics during a high current injection than did the V-LEDs. When the current was 700 mA, the *L*-*I*-*V* characteristics (Figure 5) indicated that the measured intensity of the NTG-LEDs and reference device were 298 and 252 mW, respectively. When the current was continually increased to 1,500 mA, the power difference increased to 118 mW (548 mW for NTG-LEDs and 430 mW for V-LEDs). Although the shadowing area of the NTG-LEDs was larger than that of the reference (6.3%:3.3%), the PN electrode layout induced a more favorable current spreading and led to an efficient output power. This condition enabled the NTG-LEDs to be enhanced substantially compared with the conventional V-LEDs [27-29].

**Figure 6:**
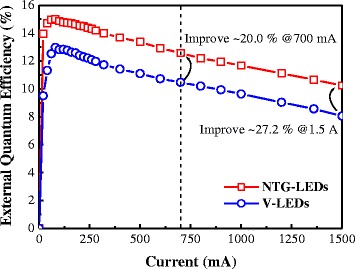
The EQE of both devices under the identical pulse current injection.

As shown in Figure 6, the external quantum efficiency (EQE) was calculated according to the *L*-*I*-*V* characteristics. The EQE of the NTG-LEDs and reference device at the same 700- and 1,500-mA current injections were 12.6% and 10.5%, and 10.3% and 8.1%, respectively. Direct comparison was performed at the same injection levels, and the results indicated that the EQEs improved by 20.0% and 27.2% at 700 mA and 1,500 mA, respectively.

**Figure 7:**
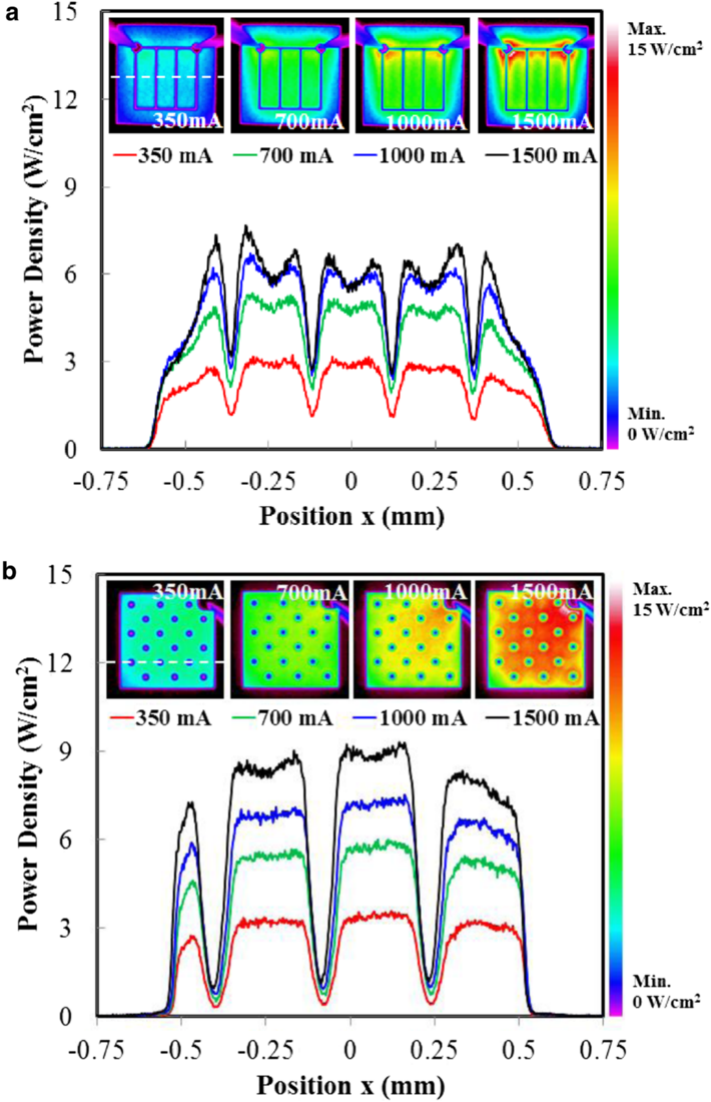
Distributed light pattern demonstrated at different current injections and relation between position *x* and power density. **(a)** V-LEDs and **(b)** NTG-LEDs.

**Figure 8:**
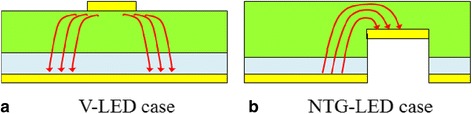
The illustration of current spreading paths in different devices: **(a)** V-LED and **(b)** NTG-LED.

One of the major features of the proposed design is the improved current injection scheme. An efficient current spreading can lead to uniform light output and prevent the nonlinear crowding effect and eventual gain reduction. Figure 7 shows the light intensity distribution of the devices at various current levels. According to both the color-contoured planar map (insets) and the cross-sectional intensity profile, the NTG-LEDs exhibited a more favorable uniformity than did the V-LEDs. The basic current paths are different between the two cases, as can be seen in Figure 8. The case (a) (conventional V-LED) is more like the classical top contact structure which follows the current spreading formula [30]:

2 $$ {L}_{\mathrm{s}}=\sqrt{\frac{t{n}_{\mathrm{ideal}}kT}{\rho {J}_0e}} $$

where *L*_s_ is the characteristic spreading length, *t* is the thickness of the n-layer in our case, *n*_ideal_ is the idealty factor of the diode, and *J*_0_ is the current density at the edge of the metal contact.

On the other hand, the arrangement of electrodes in the case (b) (the NTG-LED) is very similar to the way we model the current crowding, and the corresponding spreading length (*L*_*s*_) is:

3 $$ {L}_{\mathrm{s}}=\sqrt{\frac{\left({\rho}_{\mathrm{c}}+{\rho}_{\mathrm{p}}{t}_{\mathrm{p}}\right){t}_{\mathrm{n}}}{\rho_{\mathrm{n}}}} $$

where *ρ*_c_, *ρ*_p_, *t*_p_, *ρ*_n_, and *t*_n_ are the p-type specific contact resistance, the resistivity and thickness of the p-type layer, and the resistivity and thickness of the n-type layer, respectively.

Comparing Equations 2 and 3, one can immediately observe the injection current dependence on Equation 2 brought by the term *J*_0_. As the injection current increases, the *L*_s_ decreases. The reduction of *L*_s_ means the uneven distribution of the current, and thus, the intensity of light is getting worse. This phenomenon can be seen in the aforementioned beam profiles. Meanwhile, in Equation 3, the *L*_s_ is quite constant against the injection current, which can also be observed. Numerical analysis of maximal and minimal values across 0.7 mm of the center of the devices indicated the fair comparison of intensity uniformity. As shown in Table 1, the uniformity was calculated using the equation (max − min)/(max + min). The NTG-LEDs exhibited a more favorable intensity uniformity than did the V-LEDs, particularly in high current ranges. The arrayed n-contact pattern improved the uniformity of the current injection, thus improving the resulting output light.

**Table 1:** **Data of max. power, min. power, and uniformity in Figure** 7

**Current (mA)**	**Max. power (W/cm** ^**2**^ **)**	**Min. power (W/cm** ^**2**^ **)**	***U*** **% (NTG)**	**Max. power (W/cm** ^**2**^ **)**	**Min. power (W/cm** ^**2**^ **)**	***U*** **% VTF**
350	3.508	3.166	5.1%	3.133	2.681	7.8%
700	5.873	5.337	4.8%	5.348	4.403	9.7%
1,000	7.463	6.662	5.7%	6.721	5.548	9.6%
1,500	9.224	8.178	6.0%	7.628	5.366	17.4%

In addition to light output and current spreading, the efficiency droop of the devices was evaluated. The results indicated that the peak efficiency of both devices (V-LEDs and NTG-LEDs) were 13.0% and 15.0%, respectively. The efficiency droop (*η*) can be expressed as:

4 $$ \eta =\left(\frac{\eta_{\mathrm{peak}}-{\eta}_{\exp .}\;}{\eta_{\mathrm{peak}}}\right)\times 100\% $$

where *η* is the ratio of efficiency droop, *η*_peak_ is the maximum efficiency, and *η*_exp._ is the efficiency in different experimental currents.

The calculated droop percentages for both devices (NTG-LEDs and V-LEDs) at different current levels (at 700 and 1,500 mA) were 16.2% and 18.8%, and 31.7% and 37.7%, respectively. Therefore, the efficiency droop of the NTG-LEDs was enhanced by 16.0% and 18.9%, respectively, compared with that of the reference device. A uniform current injection can be attributed to this improvement [31].

## Conclusions

In summary, this paper presents a novel flip-chip architecture for UV-LEDs, called NTG-LEDs. Compared with the conventional flip-chip structure, the configuration of the NTG-LEDs eliminated the electrode effect, increased the light extraction, and improved current spreading. According to the proposed device design, the light-output power of NTG-LEDs was further enhanced by 18.3% and 27.4% at 700- and 1,500-mA current levels, respectively. Furthermore, the EQE of the NTG-LEDs was effectively improved by 20.0% and 27.2% when 700 and 1,500 mA of current were supplied, respectively. Finally, the efficiency droop was improved by 16.0% and 18.9% at the same current level, respectively. Therefore, the proposed NTG-LED is promising for use in the next generation of UV-LEDs.

## Abbreviations

EL: electroluminescence
EQE: external quantum efficiency
ICP: inductively couple plasma
LLO: laser lift-off
LP-MOCVD: low-pressure metal-organic chemical vapor deposition
MQW: multiple quantum wells
NTG-LEDS: novel thin-GaN-based ultraviolet light-emitting diodes
UV: ultraviolet
UV-LEDs: UV light-emitting diodes
V-LED: vertical injection light-emitting diodes
